# Swimming performance in juvenile shortnose sturgeon (*Acipenser brevirostrum*): the influence of time interval and velocity increments on critical swimming tests

**DOI:** 10.1093/conphys/cox038

**Published:** 2017-06-30

**Authors:** Adam T. Downie, James D. Kieffer

**Affiliations:** 1 Australian Research Council Centre of Excellence for Coral Reef Studies, James Cook University, Townsville, 4811 QLD, Australia; 2 Department of Biological Sciences and MADSAM Sturgeon Eco-Physiology Lab, University of New Brunswick, Saint John, NB, CanadaE2L 4L5

**Keywords:** *Acipenser brevirostrum*, Critical swim speed test, exercise physiology, shortnose sturgeon, swimming methodology, UCrit

## Abstract

The most utilized method to measure swimming performance of fishes has been the critical swimming speed (UCrit) test. In this test, the fish is forced to swim against an incrementally increasing flow of water until fatigue. Before the water velocity is increased, the fish swims at the water velocity for a specific, pre-arranged time interval. The magnitude of the velocity increments and the time interval for each swimming period can vary across studies making the comparison between and within species difficult. This issue has been acknowledged in the literature, however, little empirical evidence exists that tests the importance of velocity and time increments on swimming performance in fish. A practical application for fish performance is through the design of fishways that enable fish to bypass anthropogenic structures (e.g. dams) that block migration routes, which is one of the causes of world-wide decline in sturgeon populations. While fishways will improve sturgeon conservation, they need to be specifically designed to accommodate the swimming capabilities specific for sturgeons, and it is possible that current swimming methodologies have under-estimated the swimming performance of sturgeons. The present study assessed the UCrit of shortnose sturgeon using modified UCrit to determine the importance of velocity increment (5 and 10 cm s^−1^) and time (5, 15 and 30 min) intervals on swimming performance. UCrit was found to be influenced by both time interval and water velocity. UCrit was generally lower in sturgeon when they were swum using 5cm s^−1^ compared with 10 cm s^−1^ increments. Velocity increment influences the UCrit more than time interval. Overall, researchers must consider the impacts of using particular swimming criteria when designing their experiments.

## Teaser

We measured the critical swimming speed of sturgeon using different velocity and time intervals, to evaluate the best indicator of the swimming performance. Overall, velocity increment had the greater impact on swimming performance, and researchers should consider both velocity and time intervals when measuring swimming performance of fish.

## Introduction

Of the two main methods to measure swimming performance in fish, the critical swimming test (UCrit) is still the tool most widely used by researchers (Hammer, 1995; Plaut, 2001; Kieffer, 2010). In this test, the fish is forced to swim against an incrementally increasing velocity of water until fatigue occurs. The methodology of the test has been critically evaluated (e.g. Farlinger and Beamish, 1977; Beamish, 1978; Hammer, 1995; Kolok, 1999; Plaut, 2001), and the test remains a widely used and relevant methodology of evaluating the effects of various biotic and abiotic factors on fish (Plaut, 2001; Farrell, 2008; Kieffer and Cooke, 2009). A significant literature exists on species-specific UCrits in fish (e.g. Beamish, 1978; Peake, 2004a, b; McKenzie *et al.*, 2007; Kieffer and Cooke, 2009, Table 1), and a large amount of research has focused on high performance fish, such as Salmonids, and other game fish. Most research involving fish exercise physiology is invested in Salmonids mainly due to their importance as a fishery species and the impact of dams on stock populations (Brett and Glass, 1973; Williams and Brett, 1987). Salmonids have become a physiological ‘model species’ in the context of cardiac and exercise physiology, as they frequently swim at UCrit and thus perform at or near maximum oxygen consumption (Jain *et al.*, 1997). Recently, studies using other fish are available (see Table 1 for references), such as sturgeons, mainly due to their conservation status. However, there still is a paucity of information regarding the swimming capabilities of sturgeons, when compared to the teleosts (reviewed by Peake, 2004a).

**Table 1:** The comparison of speed increment (cm s^−1^), time interval (min) and resulting UCrit (BL s^−1^ and cm s^−1^ ± SE) for various fish species

**Species**	***T***_**L**_**(cm)**	***n***	**Temperature (°C)**	**Speed increment (cm s**^**−1**^)	**Time interval (min)**	***U***_**crit**_		**Reference**
						**BL s**^**−1**^	**cm s**^**−1**^	
**Shortnose sturgeon** (*Acipenser brevirostrum*)	19.4 ± 0.1	71	10–25	5	30	1.5 ± 0.1	29.5 ± 1.3	Deslauriers and Kieffer (2011)
	7.1 ± 0.1	6	15	3	20	3.2 ± 0.2	22.3 ± 0.6	Deslauriers and Kieffer (2012b)
	16 ± 0.7	8	15–16	5	30	1.8 ± 0.1	28.7 ± 1.1	May and Kieffer (2017)
	16.4 ± 0.7	8	15–16	5	30	1.7 ± 0.1	27.2 ± 2.1	
**Siberian sturgeon** (*Acipenser baerii*)	58.4 ± 0.6	4	24	10	10	1.8	105.5	Qu *et al.* (2013)
	64.3 ± 0.9	7	24	10	10	1.7	106.3	
**Lake sturgeon** (*Acipenser fulvescens*)	13.84 ± 0.2	24	14	5	10	28.56 ± 0.61	2.07 ± 0.05	Peake *et al.* (1995)
	39.32 ± 1.2	39				38.98 ± 0.88	1.02 ± 0.03	
	115 ± 4.72	3				107.67 ± 6.97	0.94 ± 0.5	
**Amur sturgeon** (*Acipenser schrenckii*)	18.8 ± 0.3	18	20	0.25*	30	1.96 ± 0.1	36.8 ± 1.9	Cai *et al.* (2013)
**Chinese sturgeon** (*Acipenser sinensis*)	13.7 ± 2	2	16–25	10	20	2.6 ± 0.1	36 ± 5	He *et al.* (2013)
	24.5 ± 2.4	2	10–25	10	20	2.3 ± 0.1	55.5 ± 2.5	
	35.3	1	10–16	10	20	2	70	
	40.5	1	10–16	10	20	2.1	85	
**Pallid sturgeon** (*Scaphirhynochus albus*)	21.4 ± 0.3	8	20	5	30	1.7^+^	35.9 ± 1.2	Adams *et al.* (2003)
**Shovelnose sturgeon** (*Scaphirhynochus platorynchus*)	57^–^	2	16	10	15	1.79 ± 0.2	102 ± 14	Adams *et al.* (1997, 2003)
	67.2 ± 1.4^–^	3	16	10	15	1.4 ± 0.2	90.9 ± 14.8	
	19.5 ± 0.73	6	20	5	30	1.9^+^	36.9 ± 3.5	
	20.9 ± 1.3	4	10			0.93^+^	19.5 ± 4.4	
**Green sturgeon** (*Acipenser medirostris*)	4.3 ± 0.2	32	18–19	5	5	8.5 ± 0.4	35.7 ± 1.7	Verhille *et al.* (2014)
	6.5 ± 0.2	40	18–19	5	10	7.1 ± 0.2	45.3 ± 1.5	
	15.4 ± 0.6	25	18–19	10	20	2.9 ± 0.1	43.2 ± 1.3	Allen *et al.* (2006)
	22.1 ± 0.4	27	18–19	10	20	2.2 ± 0.1	48.1 ± 1.3	
	49.4 ± 0.6	53	18–19	10	30	1.2 ± 0.5	57.5 ± 2.5	Miller *et al.* (2014)
	68.3 ± 2.7	11	19	10	20	1.2 ± 0.1	79.2 ± 4.9	Mayfield and Cech (2004)
**White sturgeon** (*Acipenser transmontanus*)	8 ± 0.4	44	18–19	5	10	4.6 ± 0.2	35.3 ± 1.4	Verhille *et al.* (2014)
	34.2 ± 1.6	14	11–12.5	5	15	1.6 ± 0.05	56.4^+^	Counihan and Frost (1999)
**Coho salmon** (*Oncorhynchus kisutch*)	61.1 ± 0.9^–^	12	7.6 ± 0.1	0.15*	5–20	1.61 ± 0.02	98.2 ± 1.8	Lee *et al.* (2003)
	57.7 ± 1.4^–^	13	8.2 ± 0.7			1.68 ± 0.05	96.5 ± 1.9	
**Chinook salmon** (*Oncorhynchus tshawytscha*)	31–33	9	8–10	0.5*	10	2.13 ± 0.08	68^+^	Gallaugher *et al.* (2001)
**Sockeye salmon** (*Oncorhynchus nerka*)	7.74 ± 0.06 (0.3 years)	10	15	9.1	60	6.65	51.5	Brett (1965)
	10.03 ± 0.17 (0.7 years)	9	15	9.1	60	5.94	59.8	
	12.78 ± 0.21 (0.9 years)	42	15	9.1	60	4.16	53.2	
	18.8 ± 0.8 (1.4 years)	10	15	9.1	60	4.12	77.4	
	41.8 ± 1.13 (3.4 years)	4	15	9.1	60	3	125	
	53.9 ± 0.67 (4.4 years)	14	15	9.1	60	2.65	143	
	64.2 ± 0.7^–^	20	18 ± 0.2	0.15*	5–20	2.08 ± 0.05	132.9 ± 1.7	Lee *et al.* (2003)
	57.9 ± 1.6^–^	6	13 ± 0.2	0.15*	5–20	2.36 ± 0.06	136.8 ± 3.4	
	62.4 ± 1.6^–^	12	15.9 ± 0.2	0.15*	5–20	1.74 ± 0.05	110.4 ± 2.7	
	64 ± 0.9^–^	12	12.2 ± 0.2	0.15*	5–20	1.41 ± 0.03	89.8 ± 1.7	
	16 ± 0.17	5	2	5	30	2.5 ± 0.13	39.98 ± 2.09	Brett and Glass (1973)
**Pink salmon** (*Oncorhynchus gorbuscha*)	49.3 ± 3.8	78	12–14	10–15	30	2.4 ± 0.75	118^+^	Williams and Brett (1987)
	46.8 ± 2.4	101	12–14	10–15	30	2.2 ± 0.87	103^+^	
**Rainbow trout** (*Oncorhynchus mykiss*)	38.9 ± 0.5	5	5.5–8	0.2*	30	1.72 ± 0.08	67^+^	Jain *et al.* (1997)
	42 ± 1	2	5.5–8	0.2*	30	1.5 ± 0.11	63^+^	
	33.3 ± 0.5	4	5.5–8	0.2*	30	2.1 ± 0.06	70^+^	
	10	56	6	2.5	5	4.3^+^	43.4	Peake *et al.* (1997b)
	10	48	18	2.5	5	5.4^+^	54.4	
**Brown trout** (*Salmo trutta*)	–	6	5	30	15	1.95 ± 0.13	–	Beaumont *et al.* (1995)
	–	6	15	30	15	1.94 ± 0.1	–	
	7.8 ± 0.2	8	–	5	20	8.3^+^	65.43 ± 0.54	Tudorache *et al.* (2008)
**Pumpkinseed** (*Lepomis gibbosus*)	12.7 ± 0.27	12	20	–	60	3.01 ± 0.27	38	Brett and Sutherland (1965)
**Common carp** (*Cyprinus carpio*)	4.9 ± 0.1	8	–	5	20	8.8^+^	43.31 ± 2.15	Tudorache *et al.* (2008)
	10.7 ± 0.2	8	–	5	20	5.8^+^	62.3 ± 4.15	
	22.8 ± 3.9	8	–	5	20	3.8^+^	87.09 ± 5.24	
**Gudgeon** (*Gobio gobio*)	10 ± 0.3	8	–	5	20	5.4^+^	54.15 ± 2.01	
	12.3 ± 0.3	8	–	5	20	4.9^+^	60.17 ± 1.17	
**Stone loach** (*Barbatula barbatula*)	7.2 ± 0.5	8	–	5	20	3.9^+^	28.25 ± 0.32	
**Common roach***(Rutilus rutilus)*	4.6 ± 0.2	8	–	5	20	10^+^	45.78 ± 2.1	
	7.3 ± 0.3	8	–	5	20	8.1^+^	59.45 ± 1.27	
	15.7 ± 1.5	8	–	5	20	7^+^	110.75 ± 6.71	
**Arctic char** (*Salvelinus alpinus*)	35.5 ± 1.2	11	–	10	10	2.8^+^	100.2 ± 3	Jones *et al.* (1974)
**Mountain whitefish** (*Prosopium williamsoni*)	30.4 ± 1.5	9	–	10	10	1.4^+^	42.5 ± 6.5	
**Arctic cisco** (*Coregonus autumnalis*)	42.1	4	–	10	10	1.9^+^	80	
**Emerald shiner** (*Notropis atherinoides*)	6.5	4	–	10	10	9.1^+^	59	
**Trout spp.**	7.2	3	–	10	10	7.6^+^	55	
**Goldeneye**(*Hiodon alosoides*)	22.5	2	–	10	10	2.7^+^	60	
**Least cisco** (*Coregonus sardinella*)	29.5	2	–	10	10	2^+^	60	
**Zebra fish** (*Danio rerio*)	4.4 ± 2.5	21	28	4	5	15.5	56 ± 4.8	Plaut (2000)
	5.2 ± 0.38	17	28	4	5	12.5	43.7 ± 6.8	
**Iberian barbel** (*Luciobarbus comizo*)	15.6–50.9	60	16–21	0.75*	30	3.1 ± 0.86	81 ± 11	Mateus *et al.* (2008)
**Creek chub** (*Semotilus astromasculatus*)	12.2 ± 0.9	7	21	3.5	2	4.3^+^	53.2 ± 1.8	Tritico and Cotel (2010)
**Guppy** (*Poecillia reticulate*)	1.75 ± 0.05	37	27–29	2.9	3	13.7	23.7 ± 0.96	Nicoletto (1991)
	1.76 ± 0.05	22	27–29	2.9	3	12.8	22.6 ± 0.79	
	1.73 ± 0.05	27	27–29	2.9	3	12.4	21.3 ± 0.65	
**Atlantic silverside** (*Menidia menidia*)	6.34 ± 0.23	10	20	0.5*	2	9.7	61^+^	Hartwell and Otto (1991)
	6.34 ± 0.23	10	20	0.5*	5	9.5	60^+^	
	6.34 ± 0.23	10	20	0.5*	10	9.7	61^+^	
	6.34 ± 0.23	10	20	0.5*	15	9.2	58^+^	
	6.34 ± 0.23	10	20	0.5*	20	9.5	60^+^	
	6.34 ± 0.23	10	20	0.5*	30	8.6	54^+^	
	6.34 ± 0.23	10	20	0.5*	45	8.5	53^+^	
	6.34 ± 0.23	10	20	0.5*	60	9.1	58^+^	
**Winter flounder** (*Pseudopleuronectes americanus*)	35.8 ± 0.6	7	4	4	15	0.65 ± 0.06	23.2 ± 2.2	Joaquim *et al.* (2004)
	38.8 ± 0.8	8	10			0.73 ± 0.07	28.3 ± 2.4	
**Scalloped hammerhead** (*Sphyrna lewini*)	55	11	21.6–28.6	10	30	1.17 ± 0.21	65 ± 11	Lowe (1996)

Note: ‘–’ indicates that information is not provided in study. The length of the fish (*T*_L_; cm ± SE), sample size (*n*) and water temperature (°C) is also provided. An asterisk (*) represents a speed interval that increases per BL s^−1^. A minus sign (–) represents the length of the fish is in fork length. A plus sign (+) represents converted UCrit values (cm s^–1^ to BL s^–1^, or vice versa) by the authors of this paper.

Most sturgeon species’ populations are vulnerable (listed as either threatened or endangered) to many anthropogenic impacts on the environment, including over-fishing and dams blocking migration routes (Rochard *et al.*, 1990; Williot *et al.*, 2002; Mussen *et al.*, 2014; Verhille *et al.*, 2014; Jager *et al.*, 2016). Dam construction limits spawning grounds, causing larvae to hatch in less-ideal locations (e.g. higher salinity) in the river (Cheong *et al.*, 2006). In addition, dams also cause changes in river flows and temperature which can impact water quality (Secor *et al.*, 2002; Cai *et al.*, 2015) and thus affect swimming performance. To mitigate migration issues with respect to dams, fishways are built so migrating fish can overpass the dam. However, most fishway designs are built for Salmonids, which have different body morphologies and swim performances than sturgeons (Peake *et al.*, 1997a; Wang and Guo, 2005; Zheng *et al.*, 2010; Thiem *et al.*, 2011; Cai *et al.*, 2013). In order to encourage Salmonids and Clupeid fish to swim through the passage, fishways rely on mechanisms such as bends and obstructions to alter flow regimes (Jager *et al.*, 2016), with water velocity ranging from prolonged swimming speed, to as high as burst swimming speed (Cai *et al.*, 2015). However, sturgeons prefer swimming along a constant flow, as evident by pallid sturgeon (*Scaphirynchus albus* Forbes and Richardson, 1905) preferring migration routes with the lowest, constant flows for energy optimization (McElroy *et al.*, 2012).White sturgeon (*Acipenser transmontanus* Richardson, 1836) benefitted from fishway designs that implemented flow straighteners to provide constant flow (Jager *et al.*, 2016). Therefore, with global sturgeon populations on the decline as a result from anthropogenic influences such as dam construction, fishways need to accommodate local sturgeon populations. Recent studies have specifically focused on exercise physiology of sturgeons to provide data that might improve fishway designs (Cai *et al.*, 2013; Braaten et al., 2015; Jager *et al.*, 2016; Thiem *et al.*, 2016). Many of these studies focus on burst swimming, which may not be an important aspect of sturgeon physiology, as their anaerobic capabilities appear to be less than teleosts (Kieffer *et al.*, 2001). Peake *et al.* (1997a) found that Lake sturgeon (*Acipenser fulvescens* Rafinesque, 1817; 12–132 cm total length; *T*_L_) are incapable of high speed or burst swimming (compared to Salmonids), as evident by fatigue graphs that do not show a slope change between prolonged and burst swim speeds. A similar trend was found in juvenile shortnose sturgeon (7.07 ± 0.38 cm *T*_L_), as there was no statistically significant change in slope as the fish transitioned from prolonged to burst swimming speeds (42 cm s^−1^; 6 BL s^−1^) during an endurance test (Deslauriers and Kieffer, 2012a). In contrast, Adams *et al.* (1999) did note a slope change for juvenile Pallid sturgeon (13–20.5 cm Fork Length; *F*_L_) during endurance tests, however, unlike many sturgeons, they are found in fast flowing rivers (>40 cm s^−1^). In addition, sturgeons have retained their primitive notochord, which limits locomotor power output (Long, 1995). Recently, it has been suggested that sturgeon rely on aerobic swimming (Kieffer *et al.*, 2009), however, relative to other species of fish, it has been shown that sturgeons have low critical swimming speeds (Webb, 1986; Peake et al., 1995; Kieffer *et al.*, 2009; Deslauriers and Kieffer, 2012a, b; Cai *et al.*, 2013, 2015; Verhille *et al.*, 2014; May and Kieffer, 2017). While this lower swimming capacity of sturgeon is partially related to its body morphology (presence of scutes, heterocercal tail; Webb, 1986; Peake, 2004a; Kieffer *et al.*, 2009; Deslauriers and Kieffer, 2011; Qu *et al.*, 2013), it is uncertain whether the methodology used to swim sturgeon might influence UCrit values, and thus the swim performance of these animals. Specifically, many swimming tests were initially developed for Salmonids (Brett, 1964), and various modifications to the procedure have been adopted (Kolok and Sharkey, 1997; Farrell, 2008), in part because of differences in fish size (age), size of flume, and reduction of time to complete tests (i.e. minimizing experimental time). Thus, standardized protocols for UCrit tests are not often adhered to, and are likely to be species specific. In particular, the magnitude of the velocity increment and prescribed time interval for a given swimming period vary (Table 1), which has been shown to affect the UCrit (Farlinger and Beamish, 1977; Beamish, 1978), and thus can greatly influence the value of comparisons between studies. For species such as sturgeon, which are benthic species that modify their swimming behaviour substantially at different swimming speeds (Kieffer *et al.*, 2009; Deslauriers and Kieffer, 2012a; May and Kieffer, 2017), the choice of velocity and time intervals might be important factors that influence the overall UCrit performance (Verhille *et al.*, 2014). To date, this is not known for any sturgeon species, and understanding this may help improve design of fishways specifically for sturgeons.

The present study was undertaken to examine the relationship between the effects of time and velocity increments on the critical swimming speed of shortnose sturgeon (*Acipenser brevirostrum* LeSueur, 1818), and these studies complement some of our earlier research on swimming performance and behaviour in sturgeon (Kieffer *et al.*, 2009; Deslauriers and Kieffer, 2011, 2012a; Downie and Kieffer, 2017; May and Kieffer, 2017). The shortnose sturgeon is found along the eastern seaboard of North America, from Saint John River, New Brunswick (only Canadian population) down to the St. John’s River, Florida, and was listed as an endangered species in 1973 under the US Endangered Species Act (Kynard, 1997) and according to DFO (Department of Fisheries and Oceans, Canada) is a species at risk (Kynard *et al.*, 2016). Several field studies have noted the negative impacts of dams on shortnose sturgeon spawning sites in South Carolina (Cooke and Leach, 2004; Finney et al., 2006), North Carolina (Moser and Ross, 1995) and Connecticut (Buckley and Kynard, 1985). While the recommendation from these studies is to construct fishways in order to improve migration over dams, field studies are limited in understanding the swim performance of these animals. The use of lab-based UCrit tests that follow a standard protocol for sturgeon may better improve the conservation strategies and construction of proper fishways for these at-risk species. It is hypothesized that UCrit values will be impacted both by swimming time and velocity increments, with the prediction that large velocity increments will lead to higher critical swimming speeds, as has been shown in a previous study by Farlinger and Beamish (1977) for largemouth bass (*Microperus salmoides* Lacépède, 1802).

## Methods and materials

### Animal husbandry

Juvenile shortnose sturgeon were obtained from Acadian Sturgeon and Caviar, Inc. (New Brunswick, Canada; http://www.acadian-sturgeon.com), and housed in 208 l holding tanks (50–80 individuals per tank) which was continuously supplied with a flow-through of fresh, well aerated, dechlorinated, city water (salinity = 0 ppt; temperature = 15 ± 1°C; pH = 7) at a rate of 1 l min^−1^. Fish were fed daily to satiation with commercial Salmonid pellets (1.5 mm optimum salmonid feed; 52% crude protein, 18% crude fat, 1.2% crude fibre; www.coreyaqua.ca) each day, but were fasted for 24 h prior to the swim trials (Deslauriers and Kieffer, 2011). A photoperiod of 14 h:10 h (day:night) was maintained throughout the holding period.

### Experimental flume

The experimental flume (Aquabiotech Inc., Coaticook, Canada) measured 732 cm (length) × 50 cm (height) × 50 cm (width). The swimming test area was 155 cm length × 17 cm height of the water × 50 cm width. An acrylic flow channel was set up ahead of the swimming area to ensure laminar flow. A mesh screen was placed at the downstream end of the flume. Flume velocity was calibrated using a FLO-MATE Marsh-McBirney portable flowmeter (Model 2000) (Deslauriers and Kieffer, 2012a, b; May and Kieffer, 2017; Downie and Kieffer, 2017). Water temperature within the flume was maintained at 15°C. Dechlorinated fresh water was used for the swim trials; after every second fish swum, approximately a third of the water was drained from the flume and replaced to replenish water oxygen levels (always >9.0 mg l^−1^).

### Swimming protocol

Fish were gently removed from the holding tank using a wet net and quickly measured under water, so they were the target length for the study (18–20 cm *T*_L_; total length; tip of rostrum to tip of caudal fin). A single fish was swum in the flume at a time. Fish were placed in the flume and allowed to recover from handling from their holding tank, at flow speeds of 5 cm s^−1^ for 30 min (following methods described in Deslauriers and Kieffer, 2012a). Following this habituation period, velocity was increased in a constant stepwise progression (Brett, 1964). Time increments used were 5, 15 and 30 min; velocity increments were 5 and 10 cm s^−1^. Eight (*n* = 8) fish were used for each set of UCrit tests. Once the swimming trial was complete, the weight of the fish was recorded and it was subsequently placed in a different holding tank, so they could not be swum more than once. Critical swimming speed (UCrit) was calculated using the following formula:

$$UCrit\;\left(cm\text{​}\;s^{-1}\right)=Vf+[\;\left(T1/t\right)\times dv],$$

where Vf is the speed of the last completed interval (cm s^−1^), T1 is the time swum at the final velocity before it fatigued (min), *t* is the time increment (5, 15 or 30 min) and dv is the velocity increment (5 or 10 cm s^−1^) (Brett, 1964). UCrit is then converted to body length per second (BL s^−1^).

### Statistical analysis

Data were graphed and presented as means ± SE. The UCrit values (BL s^−1^) were compared between velocity increments (5 or 10 cm s^−1^) and time increments (5, 15 or 30 min) using two-way ANOVAs (*α* = 0.05). Data were log_10_ transformed to better approximate normality and equal variance. A Tukey’s post hoc test was used if significant differences were found. All statistics were performed using Sigma Stat 3.5 with *α* = 0.05.

## Results

**Figure 1.**
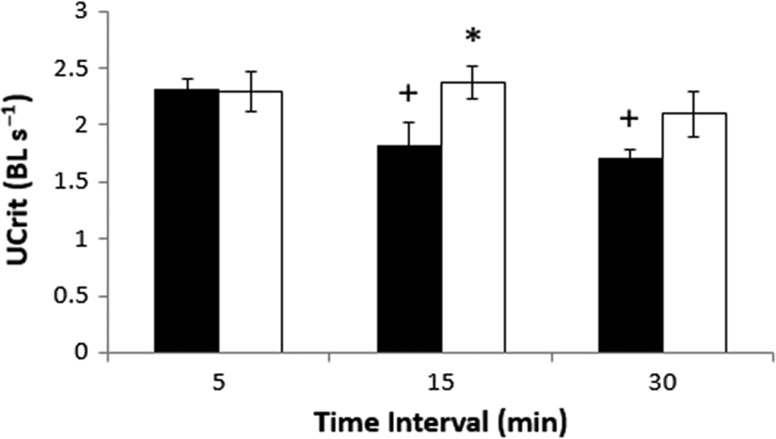
The critical swimming speed (UCrit) of juvenile shortnose sturgeon (*Acipenser brevirostrum*) swum at different speed (5 cm s^−1^; black bars and 10 cm s^−1^; open bars) and time intervals (5, 15 and 30 min). UCrit is expressed as body lengths per second (BL s^−1^). A plus sign (+) indicates a significant difference (*P* < 0.05) in UCrit values from the corresponding 5 min interval. An asterisk (*) indicates a significant difference (*P* < 0.05) in UCrit values between the different velocity increments at any given time interval. Values are means ± standard error (SE).

There were no significant differences in mass or length in fish between treatment groups (two-way ANOVA; *P* > 0.05, for both mass and length). On average, fish were 19.2 ± 0.1 cm (SE) and 22.6 ± 0.6 g (SE) across the groups. It was also observed during the swim trials that at slower water velocities (<15 cm s^−1^), sturgeon spent more time in the water column. At faster speeds (>20 cm s^−1^), sturgeon remained on the bottom of the flume (generally substrate skimming), particularly at the front of the flume. Results of a two-way ANOVA on log_10_ transformed data indicated that critical swimming (BL s^−1^) was affected by velocity increment (*P* = 0.025) and time interval (*P* = 0.046) (increment × interval interaction, *P* = 0.135; Fig. 1). Overall, the UCrit was about 20% higher in fish swum using a 10 cm s^−1^ versus 5 cm s^−1^ increment at 15 and 30 min intervals (Fig. 1). This contrasted the situation for fish swimming at 5 min intervals, where the UCrit values were nearly identical (~2.3 BL s^−^^1^) at both speed increments. When the velocity increment was set at 5cm s^−1^, critical swimming speed decreased curvilinearly with increases in time interval. In contrast, UCrit values were similar (~2.2 BL s^−1^) in fish tested at 10cm s^−1^ regardless of the interval used.

## Discussion

Farlinger and Beamish (1977) state: ‘Ideally, critical performance should be measured under conditions in which fatigue is the result of swimming, not of the method applied.’ Results from the current study show that both velocity increment and time interval affect UCrit values in shortnose sturgeon. In general, swimming fish at 10 cm s^−1^ yield higher UCrit values compared with fish swimming at 5 cm s^−1^. However, at 10 cm s^−1^, sturgeon were able to maintain consistent critical swimming speeds regardless of the time interval used. This consistency across time intervals was not evident in fish swum at 5 cm s^−1^ intervals, where the UCrit decreased curvilinearly with increases in time interval. The reason(s) for this finding may be related to cost of transport at different water velocities and/or some behavioural adjustments that might be required to swim at various speeds in sturgeon. For example, Cai *et al.* (2013) recently found that the cost of transport in juvenile Amur sturgeon (*Acipenser schrenckii* Brandt, 1869) decreased slowly with increased swimming speed, and concluded that this species of sturgeon was an efficient swimmer. Although not quantified in the present study, we noted in previous studies that sturgeon modify their behaviour when swum at various speeds during the UCrit test. Kieffer *et al.* (2009) and May and Kieffer (2017) specifically noted that shortnose sturgeon modify their swimming behaviour at various speeds by using a combination of behaviours such as station-holding, substrate skimming, and burst-and-glide behaviours. Some of these behavioural modifications have also been noted for other species of sturgeon (Adams *et al.*, 1997; Chan *et al.*, 1997; Peake, 2004a; Hoover *et al.*, 2011). To sufficiently allow a species to use specific swimming behaviours during a swimming challenge, it is essential to match swimming style with the type (e.g. flat bottom) and size of flume (Deslauriers and Kieffer, 2011). In the present study, the size of the flume was large relative to the fish size to allow for fish to modify their behaviour to match the swimming velocity and challenge, which may allow for weaker swimmers to swim at speeds approaching the UCrit values (as suggested by Deslauriers and Kieffer, 2011). Therefore, it may be possible that it takes a particular velocity (noted by May and Kieffer, 2017) and/or period of time for the fish to switch from one swimming behaviour to another. This is important as Braaten *et al.* (2015) noted that flow regimes in fishways built along the Yellowstone River (Western USA) vary throughout (flows are 1.2–2.4 m s^−1^ along the ramp of the fishway and >2.4 m s^−1^ at the crest (top) of the fishway), and thus sturgeon may or may not be able to switch swimming behaviour to cope with the change in water velocity as they swim through the passage. Coupled with this, it was noted, but not quantified, that fish swum at 5 cm s^−1^ intervals appeared to spend more time swimming in the water column, compared to fish swimming at 10 cm s^−1^. How time spent swimming in the water column versus closer to the bottom of the flume influences swimming performance (i.e. cost of transport between swimming in the water column vs swimming on the benthos) in sturgeon is not fully understood, but may be worthy of further study.

In general, our findings support earlier conclusions of Farlinger and Beamish (1977) and Beamish (1978) that a velocity increment of 10 cm s^−1^ appears to be satisfactory for swimming performance studies; however, the selection of the time interval in the various publications may be reflective of the study objectives (see Table 1). As noted by Farrell (2008), the UCrit test can be time consuming; depending on what the endpoint UCrit is used for, a shorter or modified UCrit test might be sufficient for testing the effects of abiotic and biotic factors on swimming performance. However, if the goal of the research is to couple swimming performance with metabolic costs (i.e. oxygen consumption rates), a longer time interval (greater than 20 min) is needed to ensure that the swimming fish is in steady state, and to provide enough time for multiple measurements of the oxygen consumption of the fish during swimming at each speed. However, this may be less relevant because of the enhanced oxygen measuring technology now available to researchers.

From a conservation perspective, consistent UCrit protocols will enable the aerobic swim performance of sturgeon to be accurately measured and be representative of the species under field conditions. While many swim experiments investigate swim speeds that fish can maintain over time, water velocity inside a fishway must be set at a speed that fish can make progress against (Peake *et al.*, 1995). Cai *et al.* (2013) mentioned the importance of accurate UCrit results in constructing the dimensions of fishway openings, flow of water through the passage and the number of resting pools. For threatened/endangered species, such as sturgeons, the proper construction of such structures will support local populations impacted by anthropogenic structures, such as dams. Shortnose sturgeon populations continue to be threatened by many factors, including the construction of dams which block off ideal spawning sites that cause larvae to hatch in conditions (such as salinity or temperature), which leads to lower recruitment to the adult population (Boreman, 1997; Kynard, 1997). The construction of fishways designed for the proper swim performance of these sturgeon may enable adults to over-pass dams and return to these ideal spawning sites and prevent larvae and juveniles from growing under less-ideal conditions (e.g. temperature and salinity). For example, in Connecticut, fishways have been used successfully to allow adult shortnose sturgeon to pass over the Holyoke Dam (Kynard, 1998). However, Kynard (1998) notes that most adults enter during specific flow rates into the fishway (water flow: 200–400 m^3^ s^−1^), and thus the fishways’ design have to accommodate the sturgeon’s swim performance, so the appropriate flow rates/water velocities are not too strong for the fish. Results from this study suggest that the opening of the fishway and the current flowing through it should not exceed the UCrit speed (2.2 BL s^−1^) for juvenile shortnose sturgeon (18–20 cm *T*_L_) based on the time and velocity intervals prescribed in this study.

Life stage and how swim ability changes over ontogeny are also important criteria to consider in fishway design. Juvenile green sturgeon increase UCrit from hatch until they reach a critical size when they enter seawater (Allen *et al.*, 2006). From this point onward, UCrit will either decrease or increase as the fish continues to grow, depending on season, age or thyroid hormone levels (Allen *et al.*, 2006). He *et al.* (2013) found that UCrit is higher among younger juvenile Chinese sturgeon (aged 2.5, 4.5 and 6.5 months) than older juveniles (aged 8.5, 10.5 and 12.5 months). He *et al.* (2013) hypothesized this may be because younger fish have increased muscle mass, available energy reserves and metabolic rate relative to their size early in life. Peake et al. (1995) investigated the UCrit of Lake sturgeon over ontogeny using three size classes (small fish:12–22 cm, intermediate fish: 23–55 cm and large fish: 106–132 cm *T*_L_) and found that relative critical swimming speed is higher among the smaller fish than the larger size classes. Peake *et al.* (1995) hypothesized that small fish invest more energy into growing lengthwise than girth (muscle) and as fish grow, length growth rate decreases and larger fish develop muscle mass to reduce drag. However, critical swimming speed in larger fish decreases with length as the sturgeon cannot increase enough muscle mass to overcome drag (Peake *et al.*, 1995). While studies have investigated the impact of ontogeny on behaviour of shortnose sturgeon (Richmond and Kynard, 1995; Kynard and Horgan, 2002), changes in swim speed as fish grew were not measured. Most studies investigating the swim performance of sturgeon have focused on smaller life stages (e.g. juveniles), and thus future research should also investigate the swim performance of larger juveniles or adult sturgeons, so fishways can accommodate a wider range of sizes and developmental stages. While it has been previously difficult to assess the swim speed and metabolism of large juvenile and adult sturgeon in nature, newer technology (e.g. biotelemetry) is now available to allow for such studies to be conducted (Cooke *et al.*, 2004). This is important as Braaten *et al.* (2015) state that the current fishways in use along the Yellowstone River are designed for adult pallid sturgeon (112–164 cm *T*_L_; average swimming speed in nature is 0.77–1.95 m s^−1^; flow velocity through passage is 1.2–2.4 m s^−1^) and not suitable for juveniles (average size of juvenile pallid sturgeon are 13–21 cm *T*_L_; average UCrit is 0.1–0.25 m s^−1^; average burst swimming is 0.4–0.7 m s^−1^) (Adams *et al.*, 1999). Overall, life history stage is an important metric when evaluating swim ability and sure also be considered when constructing fishways, especially near nursery grounds.

In conclusion, these findings for shortnose sturgeon support the earlier recommendations of Farlinger and Beamish (1977) and Beamish (1978) that velocity increments should range between 5 and 10 cm s^−1^, and time intervals between 10 and 30 min for UCrit tests. In a similar manner to Beamish (1978), we recommend that preliminary studies should be conducted when swimming new fish species. Once the swimming parameters have been determined for that species, researchers should adhere to them for future studies. In addition, while UCrit is a more representative measurement of Acipensarid swimming capabilities, the importance of burst swimming should also be further investigated to construct fishways that accommodate a range of swim speeds across ontogeny.
